# The impact of Cochrane Systematic Reviews: a mixed method evaluation of outputs from Cochrane Review Groups supported by the UK National Institute for Health Research

**DOI:** 10.1186/2046-4053-3-125

**Published:** 2014-10-27

**Authors:** Frances Bunn, Daksha Trivedi, Phil Alderson, Laura Hamilton, Alice Martin, Steve Iliffe

**Affiliations:** 1 Centre for Research in Primary and Community Care, University of Hertfordshire, Hatfield, Hertfordshire AL10 9AB, UK; 2 Centre for Clinical Practice, National Institute for Health and Care Excellence, Piccadilly Plaza, Manchester M1 4BD, UK; 3 Research Department of Primary Care and Population Health, UCL Medical School (Royal Free Campus), Rowland Hill Street, London NW3 2PF, UK

**Keywords:** Research impact, Cochrane collaboration, Systematic reviews, Clinical guidance

## Abstract

**Background:**

There has been a growing emphasis on evidence-informed decision-making in health care. Systematic reviews, such as those produced by the Cochrane Collaboration, have been a key component of this movement. The UK National Institute for Health Research (NIHR) Systematic Review Programme currently supports 20 Cochrane Review Groups (CRGs). The aim of this study was to identify the impacts of Cochrane reviews published by NIHR-funded CRGs during the years 2007–2011.

**Methods:**

We sent questionnaires to CRGs and review authors, interviewed guideline developers and used bibliometrics and documentary review to get an overview of CRG impact and to evaluate the impact of a sample of 60 Cochrane reviews. We used a framework with four categories (knowledge production, research targeting, informing policy development and impact on practice/services).

**Results:**

A total of 1,502 new and updated reviews were produced by the 20 NIHR-funded CRGs between 2007 and 2011. The clearest impacts were on policy with a total of 483 systematic reviews cited in 247 sets of guidance: 62 were international, 175 national (87 from the UK) and 10 local. Review authors and CRGs provided some examples of impact on practice or services, for example, safer use of medication, the identification of new effective drugs or treatments and potential economic benefits through the reduction in the use of unproven or unnecessary procedures. However, such impacts are difficult to objectively document, and the majority of reviewers were unsure if their review had produced specific impacts. Qualitative data suggested that Cochrane reviews often play an instrumental role in informing guidance, although a poor fit with guideline scope or methods, reviews being out of date and a lack of communication between CRGs and guideline developers were barriers to their use.

**Conclusions:**

Health and economic impacts of research are generally difficult to measure. We found that to be the case with this evaluation. Impacts on knowledge production and clinical guidance were easier to identify and substantiate than those on clinical practice. Questions remain about how we define and measure impact, and more work is needed to develop suitable methods for impact analysis.

## Background

In recent years, there has been a growing emphasis on the use of evidence to inform decision-making in health care [1-3], with the use of evidence seen as particularly relevant to commissioning because of the large financial commitments involved and because of the increasing complexity of health-care management decisions [4]. In addition, there has been growing interest in the way in which research is used, with researchers increasingly expected to consider the wider impacts of their work [5]. This may include the contributions research makes to health, society, culture, the economy, quality of life and public policy.

One of the key aspects of evidence-informed policy and practice has been the development of methods for the synthesis and integration of primary research, in the form of systematic reviews. Systematic reviews have been regarded as particularly important tools for decision-makers as it inherently makes sense for decisions to be based on the totality of evidence rather than a single study [6,7]. However, despite this, the extent to which policy makers and practitioners use systematic reviews as a source of evidence has been questioned [4,6]. Indeed, it has long been recognised that research may not always have the impact that researchers desire [8,9].

The Cochrane Collaboration is an independent, international organisation involved in preparing, maintaining and disseminating systematic reviews evaluating the effectiveness of health-care interventions. Cochrane systematic reviews should be uniquely placed to influence policy, practice and research as they provide a comprehensive, critical summary of what is known about effectiveness on a given topic; the rigour of their methods is widely acknowledged, and they are periodically updated in light of new evidence. However, whilst it is acknowledged that Cochrane groups produce high-quality systematic reviews [10-12], there is at present a lack of information about the impacts of Cochrane reviews.

The UK National Institute for Health Research (NIHR) systematic review programme currently supports 20 Cochrane Review Groups (CRGs) that have their editorial bases in academic or health institutions in the UK. These groups cover a broad range of health-care areas and produce almost half of all Cochrane reviews. This study was commissioned by the NIHR to identify the impacts and likely impacts of Cochrane reviews published by the 20 NIHR-funded CRGs between the years 2007 and 2011. The aims of this study were to identify impacts on clinical guidance and clinical practice and identify important gaps in knowledge and possible influence on the conduct of new primary research studies. In addition, we sought to identify barriers and facilitators to Cochrane reviews being used by guideline developers.

## Methods

**Figure 1:**
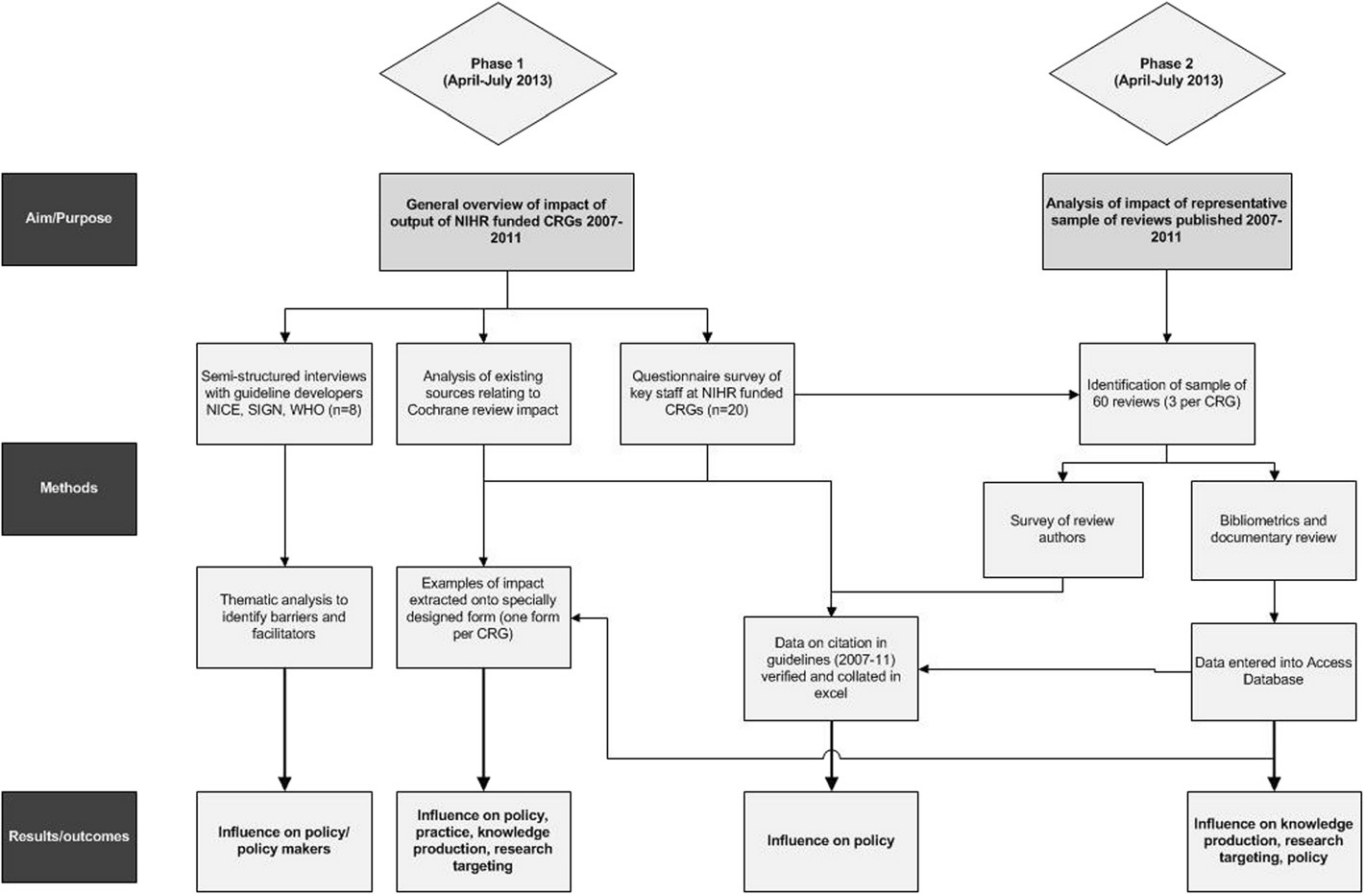
Overview of methods.

We used a mixed methods approach informed by theories about research impact [9,13-15] and guided by a framework that draws on previous work around the evaluation of research impact [16-19]. This framework, including main and subcategories, can be seen in Table 1. A variety of methods exist for evaluating research impact including bibliometrics, documentary analysis, semi-structured interviews, case studies and surveys [20,21]. As there are advantages and disadvantages of each method, it is generally recommended that a variety of sources to identify research impact are used [22,23]. We used a mixture of bibliometrics, documentary analysis, questionnaire surveys and interviews. Obtaining the ‘insider account’ has been recommended when evaluating research impact [21], and it was envisaged that staff at editorial bases and review authors would be important sources of information about review impact.There were two main components to the study. In the first (phase 1), we aimed to obtain a general overview of the impact of the outputs produced by NIHR-funded CRGs, and in the second (phase 2), we undertook a more detailed evaluation of a sample of Cochrane reviews. The study was conducted between April and September 2013 and only included reviews first published or updated during the years 2007–2011 (time period was stipulated by the funder). The evaluation covered the following CRGs: Airways; Bone, Joint and Muscle Trauma; Cystic Fibrosis and Genetic Disorders Group; Dementia and Cognitive Improvement; Depression, Anxiety and Neurosis; Ear, Nose and Throat; Epilepsy; Eyes and Vision; Gynaecological Cancer; Heart; Incontinence; Injuries; Neuromuscular; Oral Health; Pain, Palliative and Supportive Care; Pregnancy and Childbirth; Schizophrenia; Skin; Tobacco Addiction; and Wounds. For an overview of study methods, including how each part informs the findings, see Figure 1.

**Table 1:** Evaluation framework

**Main category**	**Subcategories**	**Further details**
1. Knowledge production	• Impact within research community	• Number of times review is cited
		• Stimulating debate in research community
		• Methodological developments
	• Other methods of dissemination	• Press coverage
2. Research targeting	• Influence on other research	• Identification of gaps in knowledge
		• Follow-on research
3. Informing policy development (includes actual and potential)	• Impact on national or government policy	• e.g. NICE guidance
	• Impact on international policy	• e.g. WHO guidance, or international professional bodies
	• Policies agreed at national or local level in the form of clinical or local guidelines	• e.g. Guidance produced by local trusts
	• Policies developed by those responsible for training and education	• Local or national
4. Impact on practice/services (includes actual and potential)	• Evidence-based practice	• The use of research evidence by different groups involved in clinical decision-making
		• Adoption of research findings and health technologies by health-service providers
		• Adherence to research-informed policies and guidelines
		• Addressing barriers to evidence-based practice (e.g. training)
		• Number of mentions in media
	• Quality of care	• Efficacy of health services
		• Availability, accessibility and acceptability of services
		• Utilisation and coverage
	• Cost containment and cost-effectiveness	
	• Services management and organisation	• Management of health-service procurement and provisioning (public and private)

Ethical approval for the study was obtained from the University of Hertfordshire Health and Human Sciences Ethics Committee with delegated authority (ECDA), reference number HSK/SF/UH/00003.

### Methods for phase 1: overview of impact of CRG outputs (2007–2011)

#### Questionnaire survey

We sent a questionnaire survey (Additional file 1) to the editorial base (*n* = 20) of each NIHR-funded CRG. The questionnaires were sent via email with a personalised covering letter explaining the purpose of the study. The survey included questions about general impact (both actual and potential) of the CRG output between the years 2007 and 2011; the questions were informed by our evaluation framework. Respondents were asked, where possible, to provide supporting evidence of impact. This could include citation in clinical guidelines, impact on practice (for example, changes to clinicians behaviour, changes to service organisation and delivery), or influence on future primary research. CRGs were also asked to identify reviews published (or updated) in the last 5 years that they considered to have had the most impact on policy and practice.

#### Documentary review and analysis of existing sources

In addition to the questionnaires, we hand-searched the annual reports that CRGs had provided to the NIHR Health Technology Assessment (HTA) programme for the years 2007–2012, reviewed data on the use of Cochrane reviews in National Institute for Health and Care Excellence (NICE) and Scottish Intercollegiate Guidelines Network (SIGN) guidance compiled by the UK Cochrane Centre and searched the Cochrane Quality, Innovation, Productivity and Prevention (QIPP) topics database via NHS Evidence ( https://www.evidence.nhs.uk/qipp). This documentary analysis was supplemented with keyword searches on a number of databases and internet sites including Google, NHS Evidence, and the World Health Organisation (WHO).

#### Data verification and analysis

Data from the questionnaires were entered on an Excel spreadsheet. One researcher extracted examples of actual or potential impact for each CRG and recorded this on a specially designed data extraction form based on the categories from our framework (Additional file 2). Additional data identified via documentary review was added to this form. Data relating to guidelines were collated in a separate Excel spreadsheet which was stratified by CRG. Data, and how to interpret it, was discussed with a second member of the team. Information was critically assessed and, where possible, evidence sought to verify it. For example, when a review was said to have led to follow-on research, we searched for the study protocol or final report in order to check that the review had been cited as justification for the research, and we obtained hard copies of guidelines and searched using the word ‘Cochrane’ to verify which reviews had been cited and whether they were within the time frame for this evaluation (2007–2011). We excluded examples where no supporting evidence was available.

#### Qualitative interviews

We undertook telephone interviews with a convenience sample of guideline developers; this included those involved in developing and managing guidelines and systematic reviewers and technical advisors producing evidence reviews to underpin guidance. In the UK, participants were recruited from NICE (or one of their collaborating centres) or SIGN, and international participants were recruited via the WHO.

Potential participants were identified by one of the authors with further snowballing as required. Our approach was iterative, and recruitment was stopped when we felt that we had reached data saturation. We used a semi-structured interview schedule which included questions about how systematic reviews were used in the development of guidance, experiences of using Cochrane reviews and ways in which use of Cochrane reviews might be facilitated. Interviews lasted between 20 and 40 min and were taped and transcribed. Informed consent was obtained from each participant.

#### Analysis

We drew on thematic content analysis to enable key features of guideline developers’ experiences of using Cochrane reviews to be elicited from the data [24]. To guarantee a degree of inter-rater reliability and transparency, two researchers independently scrutinised each transcript and applied open codes to text. From this, a list of initial codes and themes were created. This was refined after further discussion with the wider project team, and supporting evidence in the form of quotes was documented.

### Methods for phase 2: evaluation of impact of representative sample of Cochrane reviews (2007–2011)

In phase 2, we undertook further analysis on a purposive sample of 60 Cochrane reviews published or updated between 2007 and 2011. For each CRG, we chose one review randomly and two on the basis that they had potentially had an impact. Where possible, we used the information provided by CRGs in their questionnaires to inform the selection of reviews. However, in some cases, CRGs did not provide this information and we used other sources, such as citation counts or data from Wiley (publishers of the Cochrane Library), to guide our selection. As impact may take some time to build, we weighted our sample towards those reviews published between the years 2007 and 2010.

#### Questionnaire survey with systematic review authors

We sent a questionnaire survey (Additional file 3) to first authors of all 60 reviews. Questions were similar to those in the CRG questionnaire with authors asked to consider impact on knowledge production and possible impact of the review on health policy and practice. Authors were also asked if they thought their review identified important gaps in knowledge and/or if it had any influence on the conduct of new primary research. Methods for analysis and data verification were similar to those for the CRG questionnaire. Non-responders received a second mailing from the researchers and, in addition, 26 authors were followed up by the Managing Editors at CRG editorial bases.

#### Documentary and bibliometric analysis

We undertook citation analysis in Web of Science (WoS), Scopus and Google Scholar (searches conducted May/June 2013); undertook searches on Google, NHS Evidence and TRIP ( http://www.tripdatabase.com/) using review author and title keywords (between May and July 2013); and reviewed data from the publishers of The Cochrane Library (Wiley) on review downloads and media mentions. We also used an alternative metric measure ( http://www.altmetric.com/). This provides article level metrics which give an indication of the impact of a publication by looking at activity surrounding the publication on social media sites and in policy documents and newspapers. Articles for which no mentions have been recorded score 0. Results from the documentary and bibliometric analyses were entered into a specially designed data extraction form in Access (see Additional file 4). Data on citation counts was entered by one researcher and checked by a second.

## Results

**Figure 2:**
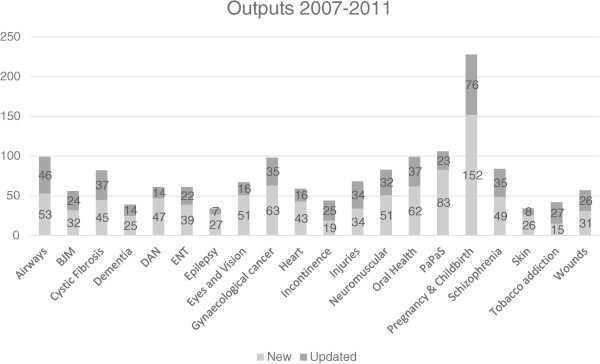
New and updated reviews published between 2007 and 2011 (stratified by CRG).

A total of 3,187 new and updated reviews were published on the Cochrane Database of Systematic Reviews between 2007 and 2011, 1,502 (47%) of which were produced by the 20 CRGs funded by the NIHR (see Figure 2).

Seventeen of the 20 (85%) CRGs and 29 (48%) of review authors returned questionnaires. There was some variation in the number of author questionnaires returned for each CRG with all three questionnaires returned for some groups and none for others. Of the 60 reviews selected for analysis, 9 (15%) were updates and the rest were new reviews. Thirteen (22%) were published in 2007, 23 (38%) in 2008, 12 (20%) in 2009 and 6 (10%) in 2010 and 2011, respectively. Thirty-four (57%) of the first authors were based in the UK. Details of these reviews, including title, country of first author and whether they were a new review or an updated review, can be seen in Additional file 5.

### Knowledge production, identifying gaps in the evidence and stimulating research

**Figure 3:**
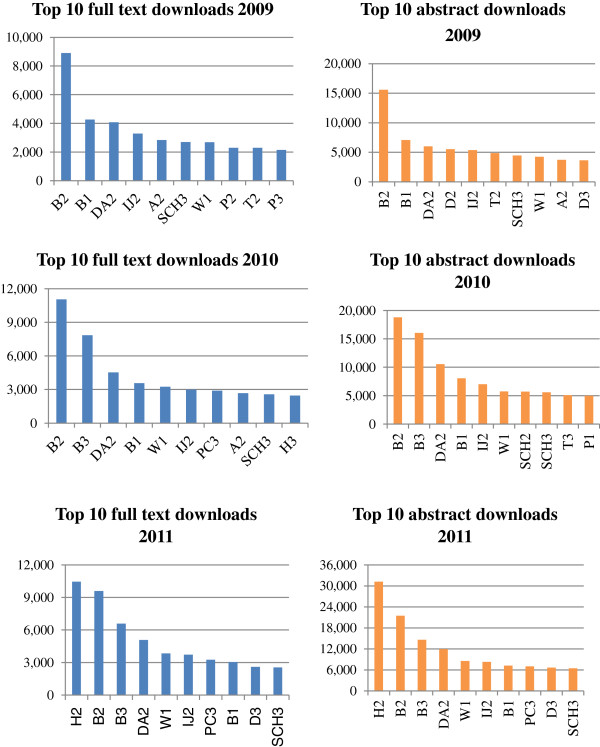
Top ten downloads from 2009 to 2011.

CRG and author questionnaires provided 40 examples where they felt reviews had influenced primary research, and 13 (22%) of the sample of 60 reviews had been cited in a protocol or the background of a primary research study. In general, most of these examples related to work conducted by the Cochrane reviewers themselves; respondents to the questionnaires were less sure if their review had influenced the research of others. Most of the examples of follow-on research were RCTs. A summary by CRG can be seen in Table 2.Although there was considerable variation between the reviews, the data do suggest that many of the 60 reviews have been of interest to decision makers. For example, 27 (45%) of the 60 reviews had had 100 or more citations in Google Scholar and 5 had received over 400 citations. Citation counts were higher in Google Scholar than in WoS or Scopus. The number of downloads from the Cochrane Library varied considerably between reviews. Of the sample of 60 reviews, the ten that were downloaded most frequently (full text and abstract) for the years 2009–2011 can be seen in Figure 3. This figure give an indication of the impact of reviews within the research and practice communities and show how downloads for reviews have increased over the 5-year period.

**Table 2:** Number of reviews informing primary research

**CRG**	**Number of reviews**	**Type and number follow-on research**	**Review is cited**^**a**^	**Type of funder**
Airways	1	RCT	Yes	Not known
Bones, Joint and Muscle	2	2 RCTs	Yes	Government (1 UK, 1 Australia)
Cystic Fibrosis	5	5 RCTs	Yes	3 government (UK), 2 not known
Depression, Anxiety and Neurosis	2	1 RCT, 1 not known	1 Yes, 1 not known	1 government (UK), 1 not known
Eyes and Vision	1	1 Not known	Yes	1 charity
Incontinence	3	3 RCTs	2 Yes, 1 not known	3 government (UK)
Injuries	7	9 RCTs	7 Yes, 2 not known	7 government (3 Australia, 3 UK, 1 Denmark), 1 industry, 1 charity
Neuromuscular	4	3 RCTs, 1 not known	2 Yes, 2 not known	2 government (1 USA, 1 France), 1 charity, 1 not known
Oral	1	RCT	Yes	1 government (UK)
Pregnancy	1	Qualitative	Yes	Not known
Schizophrenia	1	1 RCT, 2 not known	Not known	Not known
Skin	5	5 RCTs, 1 not known	4 Yes, 2 not known	3 government (UK), 2 charity (2 UK, 1 USA)
Tobacco Addiction	2	3 RCTs	Yes (all)	2 government (UK), 1 not known

^a^This relates to whether we found a study protocol or publication that cited the original Cochrane review.

Thirty-six (60%) of the 60 reviews had an Altmetric score of 1 or more with 12 (20%) having a score over 10, and 4 (7%) scoring over 50. Currently, mid-tier publications might expect 30%–40% of papers to have a score of at least 1 [25]. Although these scores need to be interpreted cautiously, for example, they may not accurately reflect the interest in reviews published before 2011, they do provide some indication of the interest around a review and the way Cochrane reviews may have impacted on knowledge production by stimulating discussion and debate [26]. Citations counts and Altmetric scores for each review can be seen in Table 3 with a summary of citation analysis data in Table 4.

**Table 3:** Number of citations (WoS, Scopus and Google Scholar) and Altmetric score

**CRG**	**Review ID**^**a**^	**Year of publication**	**Downloads 2011 (full text)**	**WoS**	**Scopus**	**GS**	**Altmetric score**
Airways	A1	2009	477	35	35	59	6
	A2	2009	2,195	49	80	168	6
	A3	2011	1,760	4	9	41	0
Bone, Joint and Muscle	B1	2007	3,047	66	123	248	8
	B2	2009	9,602	348	467	737	77
	B3	2010	6,581	96	42	242	39
Cystic Fibrosis	C1	2009	939	10	8	25	0
	C2	2008	356	4	12	19	0
	C3	2007	274	15	18	43	0
Dementia and Cognitive Improvement	D1	2008	1,660	37	59	100	1
	D2	2009	705	58	104	147	2
	D3	2007	2,599	42	104	185	2
Depression, Anxiety and Depression	DA1	2007	1,019	8	10	24	0
	DA2	2008	5,080	138	235	441	51
	DA3	2008	1,134	51	83	276	1
Ear, Nose and Throat	ENT1	2008	313	9	11	18	1
	ENT2	2007	971	56	103	168	11
	ENT3	2007	725	121	260	338	1
Epilepsy	E1	2008	147	7	19	32	0
	E2	2011	427	1	1	10	2
	E3	2008	1,187	17	40	100	0
Eyes and Vision	EV1	2007	367	1	11	45	0
	EV2	2009	417	0	2	65	0
	EV3	2009	704	12	21	34	3
Gynaecological Cancer	GC1	2007	364	23	28	64	1
	GC2	2011	295	53	21	129	0
	GC3	2008	383	28	48	61	0
Heart	H1	2008	618	16	47	95	13
	H2	2011	10,453	54	65	282	34
	H3	2010	2,440	56	75	225	1
Incontinence	IN1	2007	215	10	10	15	0
	IN2	2011	449	4	2	5	0
	IN3	2010	774	6	5	11	0
Injuries	IJ1	2008	127	7	11	13	0
	IJ2	2007	3,720	86	201	735	106
	IJ3	2007	1,373	81	188	595	0
Neuromuscular	NM1	2009	222	8	7	9	0
	NM2	2008	414	14	29	46	1
	NM3	2008	761	57	127	305	0
Oral Health	O1	2008	489	2	5	6	0
	O2	2007	413	2	15	14	11
	O3	2010	1,276	25	15	103	13
PaPaS	P1	2009	1,589	58	103	151	7
	P2	2008	2,238	27	86	139	4
	P3	2008	2,200	93	176	276	69
Pregnancy and Childbirth	PC1	2008	569	13	27	37	0
	PC2	2008	323	11	16	34	0
	PC3	2010	3,258	29	16	499	37
Schizophrenia	SCH1	2008	222	5	7	20	3
	SCH2	2010	1,666	7	16	74	1
	SCH3	2008	2,560	30	59	103	2
Skin	SK1	2007	624	14	29	67	0
	SK2	2009	327	5	10	16	0
	SK3	2009	581	16	19	70	0
Tobacco Addiction	T1	2008	903	42	89	162	1
	T2	2009	762	40	61	126	2
	T3	2010	1,481	33	31	123	17
Wounds	W1	2008	3,487	12	27	51	0
	W2	2011	123	3	0	5	4
	W3	2008	1,088	37	43	102	3

^a^For each CRG, the first review listed was chosen randomly and the other two were on the basis they may have had an impact.

**Table 4:** Summary of citation analysis data from WoS, Scopus and Google Scholar

	**WoS**	**Scopus**	**Google scholar**
Total number of citations (all 60 reviews combined)	2,192	3,562	8,333
Mean number of citations	36.5	59.3	138.8
Median number of citations	20	28.5	72
Interquartile range	7–51	11–80	25–168
Variation in counts	0–348	0–467	5–737

### Informing policy development

Systematic reviews from all the 20 CRGs were cited in some form of clinical or practice guidance. Across the CRGs, there were 722 citations in 248 guidelines (or in the evidence reviews used to develop the guidance) with 481 systematic reviews being cited at least once. Of these, 62 were international guidance, 175 national guidance, and 10 local guidance (e.g. at Trust or hospital level). Of the national guidance, 87 were developed in the UK, with Cochrane reviews cited in 30 sets of NICE guidance and 23 sets of SIGN guidance. Information on inclusion in guidelines (by CRG) is summarised in Table 5.

**Table 5:** Summary of information relating to inclusion of reviews in guidelines (stratified by CRG)

**CRG**	**Total number of citations**	**Number of reviews cited in guidelines/guidance**	**Total number of guidelines**	**Level of guideline**		
				**International**	**National**	**Local**
Airways	54	45	16	3	13	0
BJM	18	12	9	0	8	1
Cystic Fibrosis	16	15	13	3	9	1
DAN	84	52	26	10	16	0
Dementia and Cognitive Improvement	14	14	5	1	4	0
ENT	33	21	17	3	14	0
Epilepsy	8	7	3	1	2	0
Eyes and Vision	8	6	6	0	3	3
Gynaecological Cancer	7	7	3	0	3	0
Heart	37	19	26	5	21	0
Incontinence	29	22	9	1	8	0
Injuries	42	29	18	8	10	0
Neuromuscular	8	6	7	5	2	0
Oral Health	47	17	25	1	20	4
PaPaS	48	33	20	4	15	1
Pregnancy and Childbirth	129	85	33	15	18	0
Schizophrenia	57	43	8	8	6	0
Skin	20	14	14	3	11	0
Tobacco Addiction	22	18	7	0	7	0
Wounds	41	25	11	1	10	0

We interviewed eight participants, four from NICE (or NICE collaborating centres), two from SIGN and two from the WHO. Five of the interviewees were female and three were male. Two participants were senior managers involved at a strategic level in co-ordinating guideline development, and six were involved in the production of individual guidelines either as managers, systematic reviewers or technical advisors. More details of the participants and their roles can be seen in Table 6. Analysis of the interview transcripts resulted in six overarching themes and a number of subthemes relating to the views and experiences of guideline developers and their use of Cochrane reviews. The overarching themes are the following: the process of using Cochrane reviews in the development of guidance, the quality of Cochrane Reviews, culture and approaches, up-to-date evidence, methodological issues and collaboration and communication. These themes and subthemes can be seen in Table 7. Whilst a number of the issues that arose might be applicable to systematic reviews in general, the focus of our questions and analysis was on the use of Cochrane reviews.

**Table 6:** Details of interview participants

**Organisation**	**Number of participants**	**Role**
NICE - internal	2	Technical analyst, clinical guidelines team × 2
NICE - external collaborating centres	2	Senior systematic reviewer × 2
SIGN	2	Evidence and information scientist × 1
		Programme manager × 1
WHO	2	Senior manager × 2

**Table 7:** Results of thematic analysis and barriers and facilitators to the use of Cochrane reviews in the development of guidance

**Themes and subthemes**	**Barriers and facilitators**
Theme 1. The process of using Cochrane reviews (CRs) in the development of guidance	
• CRs used early in process/used in development phase	Barriers
• Systematic reviews top of evidence hierarchy/priority over other forms of evidence	• CRs may not be available, may not fit with guideline scope
• Guideline developers (GD) will use CR if available, but not always possible—CR may not be available/may not ‘fit’	• CR may be out of date
• GD may use whole CR or parts of CR (e.g. using evidence tables)/parts used vary	Facilitators
• CRs can save GD time (e.g. using existing searches/data)	• Similar evidence hierarchy
• GD may build on work of Cochrane reviewers/existing reviews	• Cochrane processes for searching/ identifying studies seen as reliable and thorough
• GD may redo the review (depending on resources)	• Similar processes for critical appraisal
	• Structure of CR means that GD can use all or part of it
Theme 2. Quality of Cochrane reviews	
• Cochrane is a respected/trustworthy brand	Barriers
• Transparent/easy to replicate	• Quality not always good
• Robust methods	• Quality may be poorer in older reviews
• Variable quality (not all good)	Facilitators
• Perception that quality may be poorer in older reviews	• Generally respected/trustworthy brand
	• Robust methods that can be replicated
Theme 3. Culture and approaches	
• Cochrane and GD have similar attitudes towards evaluating and appraising evidence	Barriers
• Cochrane reviews routinely used to inform guideline development process	• Different time frames and resources
• Some differences in methods (e.g. CR double data extraction but some GD not)	• Different priorities of Cochrane and GD
• Role of judgement (part of guideline development process but not CR)	• Different needs and perspectives
• Cochrane and GDs may have different scopes/focus/drivers behind review questions	Facilitators
• Tensions between different perspectives and interests (e.g. academic/clinical/policy)	• Similar attitudes towards evaluating and synthesising evidence
• Resources—different time frames and sources of funding	• Cochrane embedded in culture of guidelines
Theme 4. Up-to-date evidence	
• CRs can be out of date (become out of data quickly)	Barriers
• Some confusion around dates of updates	• Cochrane too slow to update
• Some GD (e.g. WHO) work with CRGs to update reviews (they fund this)	• Lack of resources to fund reviews/updates
• Delay in publication/updating	• Slow editorial processes
	Facilitators
	• Guideline developers fund CRG to update review
Theme 5. Methodological issues	
• Newer is better (newer CRs seen as methodologically better)	Barriers
• May be statistical issues (wrong data/statistical methods—barrier to use)	• Statistical issues (e.g. CR not used outcome measures, statistics GD want)
• Lack of clarity on which follow-up data used from papers	• Need for network meta-analysis and comparative analysis reviews
• Network meta-analysis, comparative analysis reviews	• Lack of facilities for sharing data
• GRADE (NICE have to use it, Cochrane do not)	
• Cochrane focus on RCTs—not always appropriate, particularly for public health	
• GD want better facilities for sharing and reanalysing data from CRs	
Theme 6. Collaboration/communication	
• Good communication improves use of review	Barriers
• Timing of communication is important	• Problems communicating with review authors and CRGs
• Dialogue/clear communication/negotiation important with appropriate persons	• Issues of ownership and authorship
• Collaboration and positive engagement might help speed things up	Facilitators
• Close collaboration between WHO and certain Cochrane groups	• Good communication between GD and authors or CRGs improves use of CR (timing important)
• Formal links between CRG and guideline developers to promote use of CR	• Financial support
• GD experience problems communicating with CRGs	
• Issues of ownership/authorship—recognition and reward	

Results from the semi-structured interviews suggest that searching for relevant Cochrane reviews is part of the guideline development process and that Cochrane reviews often play an instrumental role in informing guidance. Cochrane reviews appeared to be used at a number of different stages of the guideline development process, for example, being used early in the process to scope review questions and assess the strength of the evidence and later in the process as part of the evidence review to develop the guidance. Even when the whole Cochrane review was not used, guideline developers often drew on component parts of the review such as search strategies, lists of included and excluded studies, quality assessment data and analyses. However, there were a number of barriers to the use of Cochrane reviews in guidance. Cochrane reviews might not be available, they might not fit with the guideline scope, they may be out of date, or the methods used may not fit with those required for the guideline (see Table 7). Evidence to support the thematic analysis can be seen in Additional file 6.

### Impact on clinical practice and services

There was evidence to suggest that some Cochrane reviews may have contributed to a number of benefits to the health service including safer or more appropriate use of medication or other health technologies or the identification of new effective drugs or treatments (see the list below). Eight CRGs and 12 authors gave examples of impact on practice or services. However, it was difficult to verify some of these impacts and to judge whether these changes were directly attributable to the Cochrane review(s). Eighty-three percent of review authors who responded to the questionnaire were unsure if their work had changed the behaviour of practitioners, managers or members of the public, or if their work had helped reduce costs (69%), increase quality (76%), improve effectiveness (76%) or promote equity (79%).

• A review on support surfaces for pressure ulcer prevention [27] was used to inform guidance on purchasing within the NHS [28].

• Reviews on long-acting beta-antagonists (LABA) in asthma [29-31] may have led to safer prescribing of these drugs for people with asthma ( http://www.fda.gov/Drugs/DrugSafety/PostmarketDrugSafetyInformationforPatientsandProviders/ucm200776.htm).

• A review on colloids versus crystalloids for fluid resuscitation [32,33] may have influenced calls to stop starch use within the NHS, a decision that has the potential to save both lives and money [34].

• An updated review on antiviral treatment for Bell’s palsy [35] may have contributed to changes in practice and a reduction of prescriptions of antiviral drugs for Bells palsy ( http://cks.nice.org.uk/bells-palsy).

• A review on antifibrinolytic drugs for trauma patients [36,37] led to follow-on research which influenced the decision by the Medicines Innovation Scheme to fast track tranexamic acid for use in the NHS. Ambulance crews throughout the NHS now administer tranexamic acid to bleeding trauma patients ( http://www.swast.nhs.uk/txa.htm).

Potential benefits of Cochrane reviews were highlighted in the NICE Cochrane Quality and productivity topics, 19 of which related to reviews produced by one of the 20 CRGs during the years 2007–2011. Potential benefits identified included economic benefits through budget savings or the release of funds, improvements in clinical quality, the reduction in the use of unproven or unnecessary procedures and improvements in patient and carer experiences.

## Discussion

### Summary of findings

There was evidence that reviews from all the CRGs had had some impact. The clearest impacts were on health-care policy, with less evidence of direct impact on research targeting, clinical practice and the organisation and delivery of NHS services. From our sample of 60 reviews, there was evidence to suggest that some reviews have had a significant impact on the research and practice communities, whilst others appear to have had little or no impact (a summary of the main impacts of the 60 reviews can be seen in Additional file 7). Some of the reviews associated with the clearest evidence of impact had been updated during 2007–2011 rather than first published during that time [33,38,39].

There was considerable variation in evidence of impact between CRGs and between reviews. Variation between CRGs might be accounted for by differences in the scope or speciality of the group, the type and number of outputs, or methods used for dissemination and knowledge transfer. However, it may also be a reflection of whether CRGs routinely collect data on impact. Variation amongst the sample of 60 reviews might be a reflection of the relevance of the review findings to decision makers, the date of publication, the strength of the evidence or the strategies used to disseminate the findings.

### Knowledge production and research targeting

We found evidence to suggest that some Cochrane reviews had played a role in identifying gaps in the evidence and stimulating new research. Across the CRGs, there were 40 examples of reviews influencing primary research. However, most of these examples of research impact related to work conducted by the Cochrane reviewers themselves; there was less evidence of a broad impact on stimulating new research.

Based on the assumption that influential or important work will be cited more frequently than others [40] and that alternative metrics give an indication that papers have been read and discussed [26], the results of the bibliometric analysis suggest that a number of the reviews had had an impact on the creation of new knowledge and the stimulation of discussion and debate. Citation counts were much higher in Google Scholar than in WoS or Scopus. Google Scholar may be of particular importance to citation analyses for Cochrane reviews as previous work [41,42] suggests that citation counts for Cochrane reviews are artificially low in ISI databases and Scopus because citing authors have incorrectly referenced Cochrane reviews. However, as there are also some concerns about the accuracy of Google Scholar [43], evaluations of citation data for Cochrane reviews should include more than one database.

### Informing policy development

Reviews from all the CRGs were cited in some form of clinical or practice guidance. Mostly, this was national and international guidance, with only ten examples of local guidance. This may be because local guidance is often not available outside of the organisations involved and so may be more difficult to find. CRGs and review authors gave us a number of anecdotal examples of reviews influencing local guidance (for example, at hospital or department levels), but most were excluded from our final numbers as we were unable to find a copy of the guidance in order to verify that it referenced a Cochrane review. It is possible, therefore, that the impact of Cochrane reviews on local guidance is underestimated in our evaluation.

The fact that a review is cited in a guideline does not mean that it was instrumental in the development of the final guidance. Conversely, a Cochrane review might have been used to inform policy development but may not be referenced or cited. Interviews with guideline developers suggest that Cochrane reviews are routinely used to inform the guideline development process. However, reviews with a narrow focus or that are out of date are of less use to decision maker.

### Impact on clinical practice and services

Review authors who responded to the questionnaire were generally unsure if their work had changed the behaviour of practitioners, managers or members of the public, or if their work had helped reduce costs, increase quality, improve effectiveness or promote equity. CRGs and authors did provide some examples to suggest that Cochrane reviews had contributed to a number of benefits to the health service including safer or more appropriate use of medication or other health technologies or the identification of new effective drugs or treatments, or that they had the potential to lead to cost-saving and health-service benefits. However, attributing particular behaviour changes, health benefits or cost saving to a particular systematic review (or reviews) is difficult. Generally, new research adds to an existing pool of knowledge [44] and many research projects may lie behind a specific advance in health care [45]. Many Cochrane reviews have a narrow focus, often deliberately. Whilst, this may ensure internal validity, it may reduce its impact in the policy and practice arenas, a factor recognised in the move by the Cochrane Collaboration to introduce overviews of reviews [46].

### Strengths and limitations

Evaluating the impact of research is complicated. At present, there are no agreed instruments or methods for determining impact [47], and it is acknowledged that knowledge production is more easily discernible than impact on policy or health gain [21]. Moreover, although health benefits and broader economic benefits may be viewed as the real ‘payback’ from health research, these are hard to measure as it is difficult to attribute particular health gains to specific pieces of research [23]. Although we were able to make some inferences about health and economic benefits, these were largely beyond the remit of this study.

Our approach included the use of citation analysis and documentary review. Bibliometric techniques have been criticised for focusing on quantity rather than quality, and measuring the number of research outputs rather than research outcomes or impact [20]. Moreover, it may take several months or even years for a work to be first cited. However, we combined traditional citation counts with the use of alternative metrics. This gave us another measure of the amount of attention a review had received including its impact in social media. In addition, our mixed methods approach, including documentary review, questionnaires with CRGs and review authors, and interviews with guideline developers, enabled us to get a more complete picture of impact.

We interviewed only eight guideline developers, six of whom were based in the UK, and all of whom came from leading guideline development agencies. It is possible that their views and experiences may not be directly transferable to other organisations or to guideline developers in other countries. However, we found consistent and recurring themes in the interviews, and the views expressed are concordant with literature in this area [48,49].

We examined the impact of three reviews per CRG. Our sample is relatively small, and it is possible that the reviews we chose are not typical of the outputs of those CRGs, that there are better examples of impact and that the findings are not generalizable. Moreover, only 48% of the authors responded to the survey which raises further questions about the generalizability of the findings. However, this response rate compares favourably with previous surveys of Cochrane authors [50].

Although most of the data in the report are presented stratified by CRG, direct comparisons between reviews or between CRGs may not be appropriate. The diverse nature of the review topics and the differences in the number of questionnaires returned for each group and in length of time since publication mean that one should be cautious when comparing the impact of different reviews. Citation volume typically peaks in the third or fourth year post-publication, and therefore, a window of 5 years has been suggested as most appropriate for research assessment [51,52]. Reviews published more recently may not yet have had time to impact on policy or practice. Indeed, it has been suggested that it may take up to a decade for the full impact of research to be apparent [53].

There are several potential sources of bias in this evaluation. This was a retrospective analysis and as such may be at greater risk of bias than one where data is collected prospectively. In addition, much of our data was obtained from questionnaires to CRGs and review authors, and there is a risk of recall bias or that respondents might have inflated the impact of their work. In order to overcome this, the research team critically assessed all information provided and sought evidence to verify impacts and check whether it was related to an output produced in the time frame of interest.

### Implications of the findings

Our study provides evidence that Cochrane reviews produced by NIHR-funded CRGs have an impact on clinical guidance development and may have influenced the conduct of primary research. We found limited evidence that Cochrane reviews had had a direct effect on clinical practice, but they may have an indirect impact on health care and patient outcomes through their role in informing further primary research and clinical guidance. Although the implementation of NICE guidance has been shown to be variable [54,55], there is evidence that clinical guidelines can be effective in changing the process and outcome of care [56-60].

There are significant difficulties associated with determining the impact of specific pieces of research [21]. The focus of this evaluation was on the outputs of 20 CRGs covering a broad range of health-care topics. It is possible that it would be easier to determine impact on clinical practice and the behaviour of health-care providers if such evaluations had a narrower focus. This might allow the use of more qualitative and quantitative methods targeted at specific groups of health-care providers.

Interviews with guideline developers suggest that Cochrane reviews are routinely used in the guideline development process. However, reviews with a narrow focus or that are out of date are of less use to decision makers. Use of Cochrane reviews in the development of guidelines might be facilitated by better collaboration and communication between CRGs and guideline developers. Formal as well as informal networks might be needed to facilitate the transfer of research knowledge to decision makers [61].

## Conclusions

This study identified a number of impacts and likely impacts of Cochrane reviews. The clearest impacts of Cochrane reviews were on health-care policy, with less evidence of a direct impact on clinical practice and the organisation and delivery of NHS services. Whilst it is important for researchers to consider how they might increase the influence of their work, such impacts are difficult to measure. Questions remain about how we define and measure impact, and more work is needed to develop suitable methods for evaluating the impact of systematic reviews.

## Competing interests

FB, PA, DT and SI are all authors on Cochrane reviews. FB and DT are editors of the Cochrane Injuries Group. LH and AM declare that they have no competing interests.

## Authors’ contributions

FB, DT, PA and SI wrote the protocol. FB, DT, LH and AM analysed the data. FB wrote the first draft of the paper. PA, SI and DT contributed to the writing of the paper. All authors critically reviewed the manuscript and agreed the final version.

## Supplementary Material

Additional file 1 **CRG questionnaire.** Questionnaire for Cochrane Review Groups funded by NIHR HTA Systematic Review Programme. Click here for file

Additional file 2 **Data extraction form 1.** Questionnaire data extraction form. Click here for file

Additional file 3 **Author questionnaire.** Questionnaire for authors of Cochrane Systematic Reviews. Click here for file

Additional file 4 **Data extraction form 2.** CRIS Data extraction form for documentary and bibliometric analysis. Click here for file

Additional file 5 **Reviews selected for further analysis.** Details of 60 reviews selected for further analysis. Click here for file

Additional file 6 **Review impact.** Summary of main impacts of 60 reviews selected for further analysis. Click here for file

Additional file 7 **Qualitative interview data.** This table shows the themes that arose from our qualitative analysis and the evidence to support them. Click here for file
