# Supplementary material for: The impact of Cochrane Systematic Reviews: a mixed method evaluation of outputs from Cochrane Review Groups supported by the UK National Institute for Health Research
Source: Syst Rev. 2014 Oct 27;3:125. doi: 10.1186/2046-4053-3-125 (PMC4238314; doi:10.1186/2046-4053-3-125)
Supplement: Additional file 1 — CRG questionnaire. Questionnaire for Cochrane Review Groups funded by NIHR HTA Systematic Review Programme. [file 2046-4053-3-125-S1.doc]

# Additional file 1

# An evaluation of the impacts and likely impacts of Cochrane reviews published during 2007-2011 by Cochrane Review Groups that are supported by the National Institute of Health Research (NIHR)

# Questionnaire for Cochrane Review Groups funded by NIHR HTA Systematic Review Programme

# The University of Hertfordshire, University College London and The National Institute for Health and Clinical Excellence are working together on an evaluation of the impacts and likely impacts of Cochrane Reviews published between the years 2007-2011 by Cochrane Review Groups that are supported by the National Institute of Health Research (NIHR). This evaluation has been commissioned by the NIHR HTA Systematic Reviews Programme and will inform the quinquennial review.

# The survey will seek to collect information on the actual and potential impacts of a sample of Cochrane reviews first published, or substantially updated, by NIHR funded Cochrane Reviews Groups during the period 2007 - 2011.

# The questionnaire is designed to give you an opportunity to tell us about the impact you think reviews published by your group might have had. We are primarily interested in impacts in the UK (particularly on the NHS) but if you are aware of other types of impact then please include them. We are not expecting you to search for evidence of impact but just to let us know about anything you may already be aware of.

# Please note that although we are collecting information on the impacts of individual reviews, the focus of the evaluation is the impact of the outputs of NIHR funded Cochrane Review Groups as a whole rather than the performance of the individual CRGs or review teams.

# Dr Frances Bunn is leading the research. If you have any questions about completing the questionnaire please contact Frances at 01707 286457, email: f.bunn@herts.ac.uk

# Many thanks in advance for your help.

# A. SYSTEMATIC REVIEWS CONSIDERED MOST LIKELY TO HAVE HAD AN IMPACT

1 Please state the title of your CRG

2. We would like to identify a sample of reviews that have already had an impact. Taking into consideration all reviews that your CRG first published on the Cochrane Library between the years of 2007-2011 can you give **some examples** of those that you think have had the most impact[[1]](#footnote-2)?

Please consider only those **first published** between 2007-2011 and not updated reviews

Please list **up to 5 reviews**

| Review title and year first published | **Evidence of impact** |
| --- | --- |
|
|  |  |
|  |  |
|  |  |
|  |  |
|  |  |
|  |  |

3 Taking into consideration reviews that have been **substantially updated** during the period of 2007-2011 can you give some examples of those which you think have had the most impact?

Please list **up to 5** substantially updated reviews

| Review title and year first published | **Evidence of impact** |
| --- | --- |
|
|  |  |
|  |  |
|  |  |
|  |  |
|  |  |
|  |  |
|  |  |

# B. USE OF THE SYSTEMATIC REVIEW FINDINGS IN THE RESEARCH SYSTEM

4. Are you aware of any reviews first published by your CRG between the years 2007-2011 that have generated subsequent research (e.g. contributed to successful grant applications for primary research)?

| Yes |  |  | No |  |
| --- | --- | --- | --- | --- |

This might include further research by the authors of the systematic review or research conducted by others.

5. If you answered yes to question 4 please give further details

| **Review title** | **Further details of subsequent research (e.g. project title/topic/funder/year?)** |
| --- | --- |
|  |  |
|  |  |
|  |  |

# C. USE OF SYSTEMATIC REVIEW FINDINGS IN HEALTH SYSTEM POLICY/DECISION MAKING

**NB. Questions about applications of the findings by practitioners etc form the next section**

**6.** Review findings can be used in **policy/decision making** at any **level** (e.g international, national, regional, local trust or unit, professional, administrative or managerial) of the health service. Taking into consideration all reviews that your CRG first published on the Cochrane Library between the years of 2007-2011 can you give **any examples** of how review findings have been used in policy/decision making?

Examples of the policy relevance could take many forms including: statements by policy makers; citing of the findings in a clinical guideline from a national or local professional group etc.

**Please give relevant references/evidence**

| **Review Title** | **Evidence of impact** |
| --- | --- |
|  |  |

# D. APPLICATION OF THE SYSTEMATIC REVIEW FINDINGS THROUGH CHANGED BEHAVIOUR

**7.** Taking into consideration all reviews that your CRG first published on the Cochrane Library between the years of 2007-2011 can you give **any examples** of how review findings have led to changes, either directly or through the application of research-informed policies, in the behaviour of any of the following?

Medical/allied health professionals/other providers

Health care managers

Health service users or the wider public

| **Review title** | **Impact on behaviour** | **Any supporting evidence** |
| --- | --- | --- |
|  |  |  |

**E. HEALTH/HEALTH SERVICE/ECONOMIC BENEFITS ARISING FROM THE SYSTEMATIC REVIEW FINDINGS**

# 8. Various possible health/health service/economic benefits from the application of research findings can arise. These benefits include: improved service delivery; cost savings; improved health; an increase in values considered desirable e.g. equity.

# Taking into consideration all reviews that your CRG first published on the Cochrane Library between the years of 2007-2011 can you give any examples of how review findings might have led to health/health service/economic benefits?

#

| **Review title** | **Nature of benefits** | **Any supporting evidence** |
| --- | --- | --- |
|  |  |  |

# F. COMMENTS

# 9. If you wish, please add any further information that is not covered elsewhere

|  |
| --- |

# END

# Thank you for your help

1. This might include impact on research, health system policy/decision making, behaviour, health care practice, service organisation and economics. [↑](#footnote-ref-2)
