# Supplementary material for: The impact of Cochrane Systematic Reviews: a mixed method evaluation of outputs from Cochrane Review Groups supported by the UK National Institute for Health Research
Source: Syst Rev. 2014 Oct 27;3:125. doi: 10.1186/2046-4053-3-125 (PMC4238314; doi:10.1186/2046-4053-3-125)
Supplement: Additional file 2 — Data extraction form 1. Questionnaire data extraction form. [file 2046-4053-3-125-S2.docx]

**Additional file 2: Data extraction form 1: questionnaires**

| **Name of CRG** |  |
| --- | --- |
| **Reviews chosen for further analysis** |  |
| **1. Knowledge Production** | |
| **Impact within research community** | |
| General | |
| Review 1 | |
| Review 2 | |
| Review 3 | |
| **Other methods of dissemination (e.g. press coverage)** | |
| General | |
| Review 1 | |
| Review 2 | |
| Review 3 | |
| **2. Research targeting** | |
| **Identification of gaps in Knowledge/follow on research** | |
| General | |
| Review 1 | |
| Review 2 | |
| Review 3 | |
| **3. Informing policy development** | |
| Impact | Level (e.g. local, national, international) |
| General |  |
| Review 1 |  |
| Review 2 |  |
| Review 3 |  |
| **4. Impact on practice/services** | |
| **Quality of care** | |
| General | |
| Review 1 | |
| Review 2 | |
| Review 3 | |
| **Service management & organisation** | |
| General | |
| Review 1 | |
| Review 2 | |
| Review 3 | |
| **Cost containment & effectiveness** | |
| General | |
| Review 1 | |
| Review 2 | |
| Review 3 | |
| **Evidence based practice** | |
| General | |
| Review 1 | |
| Review 2 | |
| Review 3 | |
| **Further Comments** | |
|  | |
