# Supplementary material for: The impact of Cochrane Systematic Reviews: a mixed method evaluation of outputs from Cochrane Review Groups supported by the UK National Institute for Health Research
Source: Syst Rev. 2014 Oct 27;3:125. doi: 10.1186/2046-4053-3-125 (PMC4238314; doi:10.1186/2046-4053-3-125)
Supplement: Additional file 3 — Author questionnaire. Questionnaire for authors of Cochrane Systematic Reviews. [file 2046-4053-3-125-S3.docx]

# Additional file 3

# Author questionnaire

An evaluation of the impacts and likely impacts of Cochrane reviews published during 2007-2011 by Cochrane Review Groups that are supported by the National Institute of Health Research (NIHR)

Questionnaire for authors of Cochrane Systematic Reviews

The University of Hertfordshire, University College London and The National Institute for Health and Clinical Excellence are working together on an evaluation of the impacts and likely impacts of Cochrane Reviews published between the years 2007-2011 by Cochrane Review Groups that are supported by the National Institute of Health Research (NIHR). This evaluation has been commissioned by the NIHR HTA Systematic Reviews Programme and will inform the quinquennial review.

We are collecting information on the actual and potential impacts of a sample of Cochrane reviews published by NIHR funded Cochrane Review Groups during the period 2007-2011. The survey will seek to collect information on the actual and potential impacts of a sample of Cochrane reviews first published, or substantially updated, by NIHR funded Cochrane Reviews Groups during the period 2007 - 2011. We are not expecting you to search for evidence of impact but just to let us know about anything you may already be aware of.

Dr Frances Bunn is leading the research. If you have any questions about completing the questionnaire please contact Frances at 01707 286457, email: f.bunn@herts.ac.uk

Many thanks in advance for your help.

Please complete by xx

A. PUBLICATIONS NOT PREVIOUSLY LISTED

**1.** Please list any **additional publications (other than the Cochrane Review itself)** that have resulted directly or indirectly from the review.

Include any accepted publications that are in press but not any that are only at the submitted stage. For each publication please:

| Additional Publications |
| --- |
|  |
|  |
|  |
|  |
|  |
|  |
|  |
|  |
|  |
|  |

# B. USE OF THE SYSTEMATIC REVIEW FINDINGS IN THE RESEARCH SYSTEM

| Yes |  |  | No |  |
| --- | --- | --- | --- | --- |

**2.** Has the review generated subsequent research by **any of the review authors?**

1. If so, please give details of further grants and/or research projects

| **Research project title/topic/date** | **Funder** | **Amount** |
| --- | --- | --- |
|  |  |  |
|  |  |  |
|  |  |  |
|  |  |  |

| Yes |  |  | No |  |  | Don’t know |  |
| --- | --- | --- | --- | --- | --- | --- | --- |

1. Are you aware of any ways in which your review has contributed to further research conducted **by others?**
2. If yes, please indicate.

| **Project team** | **Research project title/topic/date** | **Further Details** |
| --- | --- | --- |
|  |  |  |
|  |  |  |
|  |  |  |

C. USE OF SYSTEMATIC REVIEW FINDINGS IN HEALTH SYSTEM POLICY/DECISION MAKING

**NB. Questions about applications of the findings by practitioners etc form the next section**

1. Review findings can be used in policy/decision making at any level (e.g international, national, regional, local trust or unit, professional, administrative or managerial) of the health service.^^[[1]](#footnote-1)^^

| Yes |  |  | No |  |  | Don’t know |  |
| --- | --- | --- | --- | --- | --- | --- | --- |

Have the findings from your review already been used in any such ways?

| Yes |  |  | No |  |  | Don’t know |  |
| --- | --- | --- | --- | --- | --- | --- | --- |

1. Are there any reasons for expecting the findings to be used for future

policy/decision making? (e.g. are they being used in guidelines that are

under development)

1. If you have replied **Yes** to either **Q6 or Q7** please give details of the use and/or expected use including: the **level** (e.g. international, national, regional, local) at which policies/decisions were (or might potentially be) influenced– please give relevant references/evidence.

|  |
| --- |

D. APPLICATION OF THE SYSTEMATIC REVIEW FINDINGS THROUGH CHANGED BEHAVIOUR

1. Do you think the findings from your review have **already led** to changes, either directly or through the application of research-informed policies, in the behaviour of the following types of people^[[2]](#footnote-2)^?

Please cross (X) the appropriate box.

| **Types of people** | **Yes** | **No** | **Don’t know** |
| --- | --- | --- | --- |
| 1. Medical/allied health professionals/other providers |  |  |  |
| 1. Health care managers |  |  |  |
| 1. Health service users or the wider public |  |  |  |

1. If you replied **Yes** to **Q9** please specify: the population whose behaviour has changed and any evidence to support claims that such changes in behaviour were caused by, or related to, the review findings

|  |
| --- |

**E. HEALTH/HEALTH SERVICE/ECONOMIC BENEFITS ARISING FROM THE SYSTEMATIC REVIEW FINDINGS**

|  |
| --- |

1. Various possible health/health service/economic benefits from the application of research findings can arise. These benefits include: improved service delivery; cost savings; improved health; an increase in values considered desirable e.g. equity.

Do you think any such benefits have already arisen as the result of the application of your review findings^[[3]](#footnote-3)^?

Please cross (X) the appropriate box.

| Nature of health/health service/economic benefits | Yes | No | Don’t know | Not applicable |
| --- | --- | --- | --- | --- |
| 1. Cost reduction in the delivery of existing services |  |  |  |  |
| 1. Qualitative improvements in the process of service delivery |  |  |  |  |
| 1. Increased effectiveness of services, e.g. increased health |  |  |  |  |
| 1. Equity, e.g. improved allocation of resources at a district/hospital level, better targeting and accessibility |  |  |  |  |
| 1. Economic benefits from a healthier workforce and reduction in working days lost |  |  |  |  |

1. If you replied Yes to Q11 please specify, giving any supporting evidence, for example the nature of the benefits; how and why the benefits have accrued.

| Nature of benefits | Supporting evidence |
| --- | --- |
|  |  |
|  |  |

F. COMMENTS

1. If you wish, please describe any other consequences of the research project not already covered.

|  |
| --- |

1. Do you have any additional comments?

|  |
| --- |

END

Thank you for your help

1. Examples of the policy relevance could take many forms including: statements by policy makers; citing of the findings in a clinical guideline from a national or local professional group etc [↑](#footnote-ref-1)
2. This might include changes in clinical behaviour (e.g. changes to the use of particular drugs or treatment) or the way managers organise or commission services. [↑](#footnote-ref-2)
3. This could include impacts to either NHS or commercial sectors [↑](#footnote-ref-3)
