# Supplementary material for: The impact of Cochrane Systematic Reviews: a mixed method evaluation of outputs from Cochrane Review Groups supported by the UK National Institute for Health Research
Source: Syst Rev. 2014 Oct 27;3:125. doi: 10.1186/2046-4053-3-125 (PMC4238314; doi:10.1186/2046-4053-3-125)
Supplement: Additional file 4 — Data extraction form 2. CRIS Data extraction form for documentary and bibliometric analysis. [file 2046-4053-3-125-S4.docx]

**Additional file 4**

**CRIS Data extraction form (data extracted into ACCESS)**

*This data extraction form applies to the 60 reviews selected for further in-depth analysis*

**A. Review details**

| **A.1** Ref ID |  |
| --- | --- |
| **A.2** Review Title |  |
| **A.3** First author |  |
| **A.4** CRG |  |
| **A.5** Method of selection (e.g. random/chosen for impact) |  |
| **A.6** Number of studies included in review |  |
| **A.7** Year first published | **A.8** Year/s updated |
| **A.9** Data extracted by: | **A.10** Date of extraction: |

**B. Citation analysis**

**Web of Science (WoS)**

| **B.1** WoS searched | **Yes**  **No** |
| --- | --- |
| **B.2** Date searched |  |
| **B.3** No of citations |  |
| **B.4** Notes |  |

**Scopus**

| **B.5** Scopus searched | **Yes  No** |
| --- | --- |
| **B.6** Date searched |  |
| **B.7** No of citations |  |
| **B.8** Notes |  |

**Google Scholar**

| **B.9** GS searched | **Yes  No** |
| --- | --- |
| **B.10** Date searched |  |
| **B.11** No of citations |  |
| **B.12** Notes |  |

**C. Further citation analysis (results of Google scholar search)**

**C.1 Are any of the papers/reports that cite the review Guidelines?**

*This might include guidance produced by NICE (or similar bodies in other countries), or by professional organisations or groups of practitioners. Include anything you think might be relevant.*

**Yes  No**

**C.2 If yes please give details^[[1]](#footnote-1)^:**

| Title of guideline/date | Organisation that produced guideline | country | Notes |
| --- | --- | --- | --- |
|  |  |  |  |

**C.3 Do any of the papers/reports that cite the review appear to be follow-on research?**

**Yes  No**

**C.4. If yes please give details:**

| Reference (including title /date/Journal/volume | How does it cite review and what was the role of the review in the research | Notes |
| --- | --- | --- |
|  |  |  |

**C.5 Are there any examples of impact other than those already listed above?**

**Yes  No**

**C.6. If yes please give details:**

| Reference (including title /date/Journal/volume | How does it cite review | Type of impact (e.g. policy/practice/research other) |
| --- | --- | --- |
|  |  |  |

**D. Further searches/documentary analysis on Google**

**Please search using key word/s from review title combined with author name (search first 5 pages of records returned only)**

**Google**

| **D.1**. Google searched | **Yes  No** |
| --- | --- |
| **D.2** Date searched |  |

**D.3 Are there any reports/documents/papers/websites that provide any evidence of impact?**

**Yes  No**

**D.4 If yes please give details**

| Source (e.g. details of paper/report/website) | How does it cite review | Type of impact (e.g. policy/practice/research other) |
| --- | --- | --- |
|  |  |  |

**NHS Evidence**

| **D.5** NHS evidence searched | **Yes  No** |
| --- | --- |
| **D.6** Date searched |  |

**D.7 Are there any reports/documents/papers/websites that provide any evidence of impact?**

**Yes  No**

**D.8 If yes please give details**

| Type of document (e.g. guidelines/paper/report) | How does it cite review | Type of impact (e.g. policy/practice/research other) |
| --- | --- | --- |
|  |  |  |

**TRIP**

| **D.9** TRIP searched | **Yes  No** |
| --- | --- |
| **D.10** Date searched |  |

**D.11 Are there any reports/documents/papers/websites that provide any evidence of impact?**

**Yes  No**

**D.12 If yes please give details**

| Type of document (e.g. guidelines/paper/report) | How does it cite review | Type of impact (e.g. policy/practice/research other) |
| --- | --- | --- |
|  |  |  |

**E. Alternative metrics**

**Altmetric**

<http://www.altmetric.com/index.php>^[[2]](#footnote-2)^

| **E.1** Altmetric searched? | **Yes  No** |
| --- | --- |
| **E. 2** Altmetric score |  |

| **E. 3** Altmetric – further relevant information |  |
| --- | --- |

**Impact Story**

<http://impactstory.org/>

| **E.4**  Impact story searched? | **Yes  No** |
| --- | --- |
| **E.5** Highly discussed | **Yes  No** |
| **E.6** Highly recommended | **Yes  No** |
| **E.7** Highly saved | **Yes  No** |
| **E.8** Highly cited | **Yes  No** |

**F. Usage data** (data provided by Wiley)

NB data only available for abstract downloads for 2009-11

| F.1 Rank 2007 |  |
| --- | --- |
| F.2 Usage count 2007 |  |
| F.3 Rank 2008 |  |
| F.4 Usage 2008 |  |
| F.5 Rank 2009 |  |
| F.6 Usage 2009 |  |
| F.7 Rank 2010 |  |
| F.8 Usage 2010 |  |
| F.9 Rank 2010 Usage count 2011 |  |
| F.10 Abstract download 2009 |  |
| F.11 Abstract download 2010 |  |
| F.12. Abstract download 2011 |  |
| F.13 Press release | Yes/No |
| F.14 If yes give details |  |
| F. 15 Number of mentions in press |  |

1. If possible download pdf of relevant paper/report and download citation into EndNote [↑](#footnote-ref-1)
2. To get alternative metrics data you will need to go to the above website and download the free bookmarklet [↑](#footnote-ref-2)
