# Supplementary material for: The impact of Cochrane Systematic Reviews: a mixed method evaluation of outputs from Cochrane Review Groups supported by the UK National Institute for Health Research
Source: Syst Rev. 2014 Oct 27;3:125. doi: 10.1186/2046-4053-3-125 (PMC4238314; doi:10.1186/2046-4053-3-125)
Supplement: Additional file 5 — Reviews selected for further analysis. Details of 60 reviews selected for further analysis. [file 2046-4053-3-125-S5.docx]

**Additional file 5: Details of reviews selected for further analysis**

| **CRG** | **Review ID** | **Review title and year of publication^1^** | **New or update** | **Country of first author** | **Questionnaire**  **returned** |
| --- | --- | --- | --- | --- | --- |
| **Airways** | A1 | Educational, supportive and behavioural interventions to improve usage of continuous positive airway pressure machines for adults with obstructive sleep apnoea 2009 (2) | New | UK |  |
|  | A2 | Pulmonary rehabilitation following exacerbations of chronic obstructive pulmonary disease 2009 (1) | New | USA | √ |
|  | A3 | Phosphodiesterase 4 inhibitors for chronic obstructive pulmonary disease 2011 (5) | New | New Zealand |  |
| **Bone Joint and Muscle** | B1 | Exercise for improving balance in adults 2007(4) | New | UK |  |
|  | B2 | Interventions for preventing falls in older people living in the community, 2009 (2) | Update^2^ | New Zealand | √ |
|  | B3 | Interventions for preventing falls in older people in nursing care facilities and hospitals 2010 (1) | Update^2^ | Australia | √ |
| **Cystic Fibrosis** | C1 | Oscillating devices for airway clearance in people with cystic fibrosis 2009(1) | New | UK | √ |
|  | C2 | Duration of intravenous antibiotic therapy in people with cystic fibrosis 2008 (2) | New | UK | √ |
|  | C3 | Oral deferiprone for iron chelation in people with thalassaemia 2007 (3) | New | UK |  |
| **Dementia and Cognitive Improvement** | D1 | Physical activity programs for persons with dementia 2008(3) | New | Canada |  |
|  | D2 | Statins for the prevention of dementia 2009 (2) | Update | Northern Ireland, UK |  |
|  | D3 | Interventions for preventing delirium in hospitalised patients 2007 (2) | New | UK |  |
| **Depression, Anxiety and Depression** | DA1 | Family interventions for bipolar disorder - Justo et al 2007 (4) | New | Brazil | √ |
|  | DA2 | Exercise for depression 2008 (4) | New | UK |  |
|  | DA3 | Cognitive behaviour therapy for chronic fatigue syndrome in adults 2008 (3) | Update | UK |  |
| **Ear, nose and throat** | ENT1 | Corticosteroids as adjuvant to antiviral treatment in Ramsay Hunt syndrome (herpes zoster oticus with facial palsy) in adults; 2008 (3) | New | UK |  |
|  | ENT2 | Nasal saline irrigations for the symptoms of chronic rhinosinusitis 2007 (3) | New | UK | √ |
|  | ENT3 | Allergen injection immunotherapy for seasonal allergic rhinitis 2007 (1) | New | UK | √ |
| **Epilepsy** | E1 | Vigabatrin for refractory partial epilepsy 2008 (3) | New | UK |  |
|  | E2 | Antiepileptic drugs for treating seizures in adults with brain tumours 2011 (8) | New | UK |  |
|  | E3 | Drug management for acute tonic-clonic convulsions including convulsive status epilepticus in children 2008 (3) | Updated | UK |  |
| **Eyes and Vision** | EV1 | Laser trabeculoplasty for open angle glaucoma 2007 (4) | New | Brazil | √ |
|  | EV2 | Medical vs surgical interventions for open angle glaucoma 2009 (1) | Update | UK | √ |
|  | EV3 | Antiangiogenic therapy with anti-vascular endothelial growth factor modalities for diabetic macular oedema 2009 (4) | New | Italy | √ |
| **Gynaecological Cancer** | GC1 | Adjuvant radiotherapy for stage I endometrial cancer 2007 (2) | New | UK |  |
|  | GC2 | Intraperitoneal chemotherapy for the initial management of primary epithelial ovarian cancer 2011 (11) | Update | Australia |  |
|  | GC3 | Anticoagulation for the long term treatment of venous thromboembolism in patients with cancer 2008 (1) | New | Lebanon | √ |
| **Heart** | H1 | Hawthorn extract for treating chronic heart failure 2008 (1) | New | UK |  |
|  | H2 | Statins for the primary prevention of cardiovascular disease 2011 (1) | New | UK | √ |
|  | H3 | Structured telephone support or telemonitoring programmes for patients with chronic heart failure 2010 (8) | New | Australia |  |
| **Incontinence** | IN1 | Absorbent products for light urinary incontinence in women 2007 (2) | Update^3^ | UK |  |
|  | IN2 | Surgery for stress urinary incontinence due to presumed sphincter deficiency after prostate surgery 2011 (4) | New | Brazil |  |
|  | IN3 | Washout policies in long-term indwelling urinary catheterisation in adults 2010 (3) | New | UK |  |
| **Injuries** | IJ1 | Beta-2 receptor antagonists for acute traumatic brain injury 2008 (1) | New | UK | √ |
|  | IJ2 | Colloids vs crystalloids for fluid resuscitation in critically ill patients 2007 (4) | Update | UK | √ |
|  | IJ3 | Anti-fibrinolytic use for minimising perioperative allogeneic blood transfusion 2007 (4) | New | Canada | √ |
| **Neuromuscular** | NM1 | Drug treatment for spinal muscular atrophy type I 2009 (1) | New | Netherlands. |  |
|  | NM2 | Treatment for POEMS (polyneuropathy, organomegaly, endocrinopathy, M-protein, and skin changes) syndrome; 2008 (4) | New | Japan | √ |
|  | NM3 | Glucocorticoid corticosteroids for Duchenne muscular dystrophy 2008 (1) | Update | UK |  |
| **Oral health** | O1 | Occlusal interventions for periodontitis in adults 2008 (3) | New | UK |  |
|  | O2 | Interventions for the treatment of oral and oropharyngeal cancers: surgical treatment. 2007 (4) | New | UK |  |
|  | O3 | Fluoride toothpastes of different concentrations for preventing dental caries in children and adolescents 2010 (1) | New | UK |  |
| **PaPaS** | P1 | Psychological therapies for chronic pain (excluding headache) in adults 2009 (2) | New | UK | √ |
|  | P2 | Non pharmacological interventions for use in breathlessness in the advanced stages of malignant and non-malignant diseases 2008 (2) | New | UK |  |
|  | P3 | Exercise for the management of cancer related fatigue in adults 2008(2) | New | UK | √ |
| **Pregnancy and Childbirth** | PC1 | Fetal fibronectin testing for reducing the risk of preterm birth 2008 (4) | New | USA | √ |
|  | PC2 | Intracervical prostaglandins for induction of labour. 2008 (1) | New | Switzerland |  |
|  | PC3 | Active versus expectant management for women in the third stage of labour. 2010 (7) | New | Ireland | √ |
| **Schizophrenia** | SCH1 | Atypical antipsychotics for people with both schizophrenia and depression 2008(1) | New | UK |  |
|  | SCH2 | Exercise Therapy for Schizophrenia 2010 (5) | New | Canada | √ |
|  | SCH3 | Psychosocial Interventions for people with both severe mental illness and substance misuse 2008 (1) | Update | Australia | √ |
| **Skin** | SK1 | Psychological and educational interventions for atopic eczema in children 2007 (3) | New | UK | √ |
|  | SK2 | Safety of topical corticosteroids in pregnancy 2009 (3) | New | Taiwan | √ |
|  | SK3 | [Surgical excision margins for primary cutaneous melanoma](http://plus.mcmaster.ca/EvidenceUpdates/LFE.aspx?5POAK6JBB3P5TZIQ3E39&r=30284) 2009 (4) | New | Australia | √ |
| **Tobacco** | T1 | Mass media interventions for smoking cessation in adults 2008 (1) | New | UK | √ |
|  | T2 | Interventions for preventing weight gain after smoking cessation 2009 (1) | New | UK |  |
|  | T3 | Legislative smoking bans for reducing secondhand smoke exposure, smoking prevalence and tobacco consumption 2010 (4) | New | Ireland |  |
| **Wounds** | W1 | Risk assessment tools for the prevention of pressure ulcers 2008 (3) | New | Ireland |  |
|  | W2 | Support surfaces for treating pressure ulcers 2011 (12) | New | Australia | √ |
|  | W3 | Antibiotics and antiseptics for venous leg ulcers 2008 (1) | New | UK | √ |

1- For each CRG the first review listed was chosen randomly and the other 2 on the basis they may have had an impact
