# Supplementary material for: The impact of Cochrane Systematic Reviews: a mixed method evaluation of outputs from Cochrane Review Groups supported by the UK National Institute for Health Research
Source: Syst Rev. 2014 Oct 27;3:125. doi: 10.1186/2046-4053-3-125 (PMC4238314; doi:10.1186/2046-4053-3-125)
Supplement: Additional file 6 — Review impact. Summary of main impacts of 60 reviews selected for further analysis. [file 2046-4053-3-125-S6.docx]

**Additional file 6: Summary of main impacts for 60 selected reviews**

| **Review title and year of publication^1^** | **Knowledge production** | **Follow-on research** | **Informing policy** | **Impact on practice/services** |
| --- | --- | --- | --- | --- |
| **Airways** | | | | |
| Educational, supportive and behavioural interventions to improve usage of continuous positive airway pressure machines for adults with obstructive sleep apnoea 2009 | GS^2^ citations =59 |  | Yes (1 national) |  |
| Pulmonary rehabilitation following exacerbations of chronic obstructive pulmonary disease 2009 | GS citations =168 | Yes –RCT | Yes (6 national, 1 international) |  |
| Phosphodiesterase 4 inhibitors for chronic obstructive pulmonary disease 2011 | GS citations =41 |  |  | Phosphodiesterase 4 inhibitors no longer recommended in practice. Potential link to review. Puhan(2011) |
| **Bone Joint and Muscle Trauma** | | | | |
| Exercise for improving balance in adults 2007 | GS citations =737  Podcast on CL & press release (81 press mentions) |  | Yes (1national) |  |
| Interventions for preventing falls in older people living in the community 2009 | GS citations =737  One of top 10 most cited reviews in CL in 2011  Has led to a number of follow on publications  High number of downloads, high altmetric score | Yes – primary research  Impact on choice of outcome measures for falls reviews (not verified). | Yes (3 national, 1 local) |  |
| Interventions for preventing falls in older people in nursing care facilities and hospitals 2010 | GS citations =242  One of top 10 most cited reviews in CL in 2011, high altmetric score |  | Yes (4 national, 1 local) |  |
| **Cystic Fibrosis** | | | | |
| Oscillating devices for airway clearance in people with cystic fibrosis 2009 | GS citations =25 | Yes – RCT | Yes (2 national, 1 local) |  |
| Duration of intravenous antibiotic therapy in people with cystic fibrosis 2008 | GS citations =19 | Yes – RCT |  |  |
| Oral deferiprone for iron chelation in people with thalassaemia 2007 | GS citations =43 |  | Yes (1national) |  |
| **Dementia and Cognitive Improvement** | | | | |
| Physical activity programs for persons with dementia 2008 | GS citations =100 |  |  |  |
| Statins for the prevention of dementia 2009 | GS citations = 147 |  |  | Cochrane quality & productivity topic |
| Interventions for preventing delirium in hospitalised patients 2007 | GS citations =185 |  | Yes (1national) |  |
| **Depression, Anxiety and Depression** | | | | |
| Family interventions for bipolar disorder 2007 | GS citations =24 | Yes (details not clear) | Yes (1 international, 2 national) | Anecdotal evidence used to influence local practice (no supporting evidence) |
| Exercise for depression 2008 | GS citations =441  One of top 10 most cited reviews in CL in 2011 |  | Yes (4 national, 1 international) |  |
| Cognitive behaviour therapy for chronic fatigue syndrome in adults 2008 | GS citations =276 |  | Yes (1international) |  |
| **Ear, nose and throat** | | | | |
| Corticosteroids as adjuvant to antiviral treatment in Ramsay Hunt syndrome (herpes zoster oticus with facial palsy) in adults 2008 | GS citations =18 |  |  |  |
| Nasal saline irrigations for the symptoms of chronic rhinosinusitis 2007 | GS citations =168 |  | Yes (1 international, 4 national) |  |
| Allergen injection immunotherapy for seasonal allergic rhinitis 2007 | GS citations =338  Highly cited |  | Yes (2 international, 1 national) |  |
| **Epilepsy** | | | | |
| Vigabatrin for refractory partial epilepsy 2008 | GS citations =32 |  |  |  |
| Antiepileptic drugs for treating seizures in adults with brain tumours 2011 | GS citations =10 |  |  |  |
| Drug management for acute tonic-clonic convulsions including convulsive status epilepticus in children 2008 | GS citations =100 |  | Yes (1 international) |  |
| **Eyes and Vision** | | | | |
| Laser trabeculoplasty for open angle glaucoma 2007 | GS citations =45 |  | Yes (2 national) |  |
| Medical vs surgical interventions for open angle glaucoma 2009 | GS citations =65 |  |  |  |
| Antiangiogenic therapy with anti-vascular endothelial growth factor modalities for diabetic macular oedema 2009 | GS citations =34  Resulted in follow on paper ([97](#_ENREF_97)) |  | Yes (2 local) | Anecdotal evidence that findings of review have impacted on local clinical practice (not verified) |
| **Gynaecological Cancer** | | | | |
| Adjuvant radiotherapy for stage I endometrial cancer 2007 | GS citations =64 |  |  |  |
| Intraperitoneal chemotherapy for the initial management of primary epithelial ovarian cancer 2011 | GS citations =129 |  |  |  |
| Anticoagulation for the long term treatment of venous thromboembolism in patients with cancer 2008 | GS citations =61 | Yes – led to further Cochrane reviews | Yes (1 national) |  |
| **Heart** | | | | |
| Hawthorn extract for treating chronic heart failure 2008 | GS citations =95 |  |  |  |
| Statins for the primary prevention of cardiovascular disease 2011 | GS citations =282  High number of downloads, highly cited since publication |  | Yes (2 national) |  |
| Structured telephone support or telemonitoring programmes for patients with chronic heart failure 2010 | GS citations = 225  Podcast and press release (100 press mentions) |  | Yes (1 international, 1 national) |  |
| **Incontinence** | | | | |
| Absorbent products for light urinary incontinence in women 2007 | GS citations =15 |  | Yes (1 national) |  |
| Surgery for stress urinary incontinence due to presumed sphincter deficiency after prostate surgery 2011 | GS citations =5 |  | Yes (1International) |  |
| Washout policies in long-term indwelling urinary catheterisation in adults | GS citations =11 |  | Yes (1 international) |  |
| **Injuries** | | | | |
| Bradykinin beta-2 receptor antagonists for acute traumatic brain injury 2008 | GS citations =13 | Yes – further systematic review & further RCT | Yes (1 national) | Indirect impact – in response to follow on research commercial development of drug was stopped |
| Colloids vs crystalloids for fluid resuscitation in critically ill patients 2007 | GS citations =735  Highly cited | Yes – RCTs | Yes (1 international, 1 national) | Review (and follow on research) led to review by EMA & suspension of use of HES  Cochrane quality & productivity topic |
| Anti-fibrinolytic use for minimising perioperative allogeneic blood transfusion 2007 | GS citations =595  Highly cited | Yes – further review and further RCT | Yes (1 international, 1 national) | Potentially – may have impacted on EMA review  Potential impact on practice – has been switch away from other Anti-fibrinolytic drugs to Tranexamic acid |
| **Neuromuscular** | | | | |
| Drug treatment for spinal muscular atrophy type I 2009 | GS citations =9 |  |  |  |
| Treatment for POEMS (polyneuropathy, organomegaly, endocrinopathy, M-protein, and skin changes) syndrome. 2008 | GS citations =46  Some follow on publications |  | Yes (1 international) |  |
| Glucocorticoid corticosteroids for Duchenne muscular dystrophy. 2008 | GS citations = 305 |  | Yes (1 national) |  |
| **Oral health** | | | | |
| Occlusal interventions for periodontitis in adults. 2008 | GS citations =6 |  |  |  |
| Interventions for the treatment of oral and oropharyngeal cancers: surgical treatment. 2007 | GS citations =14 |  |  |  |
| Fluoride toothpastes of different concentrations for preventing dental caries in children and adolescents. 2010 | GS citations =103  Podcast & press release (37 press mentions) |  | Yes (7 national, 2 local) |  |
| **PaPaS** | | | | |
| Psychological therapies for chronic pain (excluding headache) in adults. 2009 | GS citations = 151  Related publication |  | Yes 1 (national) |  |
| Non pharmacological interventions for use in breathlessness in the advanced stages of malignant and non-malignant diseases. 2008 | GS citations =139 |  | Yes ( 2 national) | Cochrane quality & productivity topic |
| Exercise for the management of cancer related fatigue in adults. 2008 | GS citations =276 |  | Yes (2 national) |  |
| **Pregnancy and Childbirth** | | | | |
| Fetal fibronectin testing for reducing the risk of preterm birth. 2008 | GS citations =37 |  |  | Potential economic impact – in US health insurers may not pay for testing in asymptomatic women (anecdotal no supporting evidence) |
| Intracervical prostaglandins for induction of labour. 2008 | GS citations =34 |  |  |  |
| Active versus expectant management for women in the third stage of labour. 2010 | GS citations =499  Further review | Yes – Qualitative study | Yes (2 international) | Change in midwifery practice in Ireland (unpublished data) |
| **Schizophrenia** | | | | |
| Atypical antipsychotics for people with both schizophrenia and depression. 2008 | GS citations =20 |  | Yes (2 national) | . |
| Exercise Therapy for Schizophrenia. 2010 | GS citations =74  Podcast & press release (54 press mentions)  Number of related publications published | Yes, primary research (details not clear) |  | Impact on local practice (anecdotal) |
| Psychosocial Interventions for people with both severe mental illness and substance misuse. 2008 | GS citations =103  Number of related publications published | Yes- primary research (details not clear) | Yes (1 national) |  |
| **Skin** | | | | |
| Psychological and educational interventions for atopic eczema in children. 2007 | GS citations =67 | Yes – RCT | Yes (1 national) |  |
| Safety of topical corticosteroids in pregnancy. 2009 | GS citations =16 | Yes – primary research (details not clear) | Yes (2 national, 1 international) | Potential – reduce adverse effects |
| [Surgical excision margins for primary cutaneous melanoma](http://plus.mcmaster.ca/EvidenceUpdates/LFE.aspx?5POAK6JBB3P5TZIQ3E39&r=30284). 2009 | GS citations =70 |  |  |  |
| **Tobacco** | | | | |
| Mass media interventions for smoking cessation in adults. 2008 | GS citations =162 |  |  |  |
| Interventions for preventing weight gain after smoking cessation. 2009 | GS citations =126 | Yes – primary research (RCTs) | Yes (1 national) |  |
| Legislative smoking bans for reducing secondhand smoke exposure, smoking prevalence and tobacco consumption. 2010 | GS citations =123  Podcast & press release (49 press mentions) |  |  |  |
| **Wounds** | | | | |
| Risk assessment tools for the prevention of pressure ulcers. 2008 | GS citations =51 |  | Yes (2 national) |  |
| Support surfaces for treating pressure ulcers. 2011 | GS citations =5 |  | Yes (1 national) |  |
| Antibiotics and antiseptics for venous leg ulcers. 2008 | GS citations =102 |  | Yes (3 national) |  |
