# Supplementary material for: The impact of Cochrane Systematic Reviews: a mixed method evaluation of outputs from Cochrane Review Groups supported by the UK National Institute for Health Research
Source: Syst Rev. 2014 Oct 27;3:125. doi: 10.1186/2046-4053-3-125 (PMC4238314; doi:10.1186/2046-4053-3-125)
Supplement: Additional file 7 — Qualitative interview data. This table shows the themes that arose from our qualitative analysis and the evidence to support them. [file 2046-4053-3-125-S7.docx]

**Additional file 7: Qualitative interview data, themes and Supporting Evidence**

| **Theme and subthemes** | **Supporting evidence** |
| --- | --- |
| **1. The process of using Cochrane reviews (CR) in the development of guidance** | |
| CRs used early in process/used in development phase | *‘almost every time we start talking about evidence, the first thing being raised is is there any Cochrane reviews’* (Participant 7 referring to guideline development committee)  *‘We use them during the development phase of the guideline’,* (Participant 4 referring to systematic reviews)  *‘the first thing that we would do when we’re looking for evidence is we would look for systematic reviews that have already been published because we think there’s no point in doing, carrying out another systematic review if it’s already been done before’* (Participant 8)  *‘normally at the guideline group meetings I might present the findings (of CR)… to give them an introduction into what kind of research is out there already’* (Participant 4)  *‘we start our guideline scoping process by looking at what is available in the Cochrane library and, because that helps us to at least draw the envelope around what kind of interventions have been reviewed* ‘(Participant 2) |
| Systematic reviews top of evidence hierarchy/priority over other forms of evidence | *‘systematic reviews are the basis for guideline development’* (Participant 6)  *‘we would start off looking at systematic reviews first’* (Participant 1)  *‘our first line is that we look for systematic reviews then we look for RCT’s or diagnostic or observational’*  (Participant 8) |
| Guideline developers (GD) will use CR if available, but not always possible - CR may not be available/may not ‘fit’ | *‘If you find a couple of good Cochrane reviews you think oh thank heavens, you know Cochrane have done it. So we would use those first and foremost’* (Participant 8)  *‘If it fits the review protocol perfectly, we will just use the review as a review….. Obviously, it’s quite rare that that happens’* (Participant 7)  ‘*So we’ve used that as a basis, but our inclusion exclusion criteria was slightly different,…..but it gives us a grounding really’* (Participant 4) |
| GD may use whole CR or parts of CR (e.g. using evidence tables)/parts used vary | *‘We would build on an existing review if it directly addresses the review question and then we might just update the search to look for some new evidence’.* (Participant 5)  *‘I will look for a Cochrane review if I find one and I’ll use the search terms…* *I think it’s quite useful to use other people’s building blocks’* (Participant 1)  *‘they do quite a lot of good statistical analysis as well which we can use’*  (Participant 8 referring to Cochrane review) |
| CRs can save GD time (e.g. using existing searches/data) | *‘I wish other systematic reviews and meta-analysis were that clearly laid out, it certainly cuts down on an awful lot of leg work’* (Participant 1)  *‘Cochrane have done a search and they’ve not found any RCT’s so we’d then think well there’s no point in us going looking for RCT’s because we know already that there aren’t any’* (Participant 8)  ‘*where we know there are good existing reviews, such as Cochrane reviews, and the evidence base would be too big for us to be able to review every review question going back to RCT evidence, for example, and extracting all the data ourselves from scratch. So, in that case, we would build on an existing review if it directly addresses the review question and then we might just update the search to look for some new evidence’* (Participant 5)  *‘the search that’s done for Cochrane reviews are usually quite good and therefore you can rely on that as a source of ah, of identifying existing studies, primary level research’*. (Participant 5) |
| GD may redo the review | *‘Sometimes, you know, we find ourselves covering areas again because there’s so many differences between things* (referring to differences between guideline scope and systematic review) *…it’s faster for us to just do it the way that we want from the beginning’*  (Participant 3)  *‘Cochrane reviews tend to focus on individual treatments and so the ability to pull all that information together when it’s in different reviews is challenging and sometimes amounts to too much work in terms of it being efficient and it can be more efficient to conduct the review yourself’* (Participant 5) |
| **2. Quality of Cochrane reviews** | |
| Cochrane is a respected/trustworthy brand | *‘it’s you know, a very reliable, trustworthy brand that people know about’* (Participant 1)  *‘we always put them through a critical appraisal and you know we find them to be rigorous and trustworthy’*  (Participant 8)  *‘There might be several systematic reviews, but the Cochrane Review would be the first one that I would use in terms of process.’* (Participant 4) |
| Transparent/easy to replicated | *‘as you know, like Cochrane reviews are presented, it’s …easier than many other reviews to add new studies and re-analyse it and yes, that’s frequently done’* (Participant 6)  *‘Well I think the fact that they’re reliable and, you know, it’s very much the same template each time’* (Participant 1)  ‘*it’s transparent the method that was used’*, (Participant 5)  *‘Well in general I think the advantage is that Cochrane Reviews are much more transparent. You can easily see, you know, the roll numbers and how the quality assessment have been done and summary of findings tables, so they are much more easy to work with’* (Participant 2) |
| Robust methods | *‘Well I think they’re kind of gold dust, you know, I’m always really pleased when I find one because I think they’re good quality but they’re also laid out in a very systematic way’* (Participant 8)  *‘I guess the question is it, in the end, is it reliable enough to base to make sound judgements and it usually is, yeah, in our experience’* (Participant 5)  *‘we know the search standards are very high’* (Participant 2) |
| Variable quality (not all good) | *‘I think the majority of Cochrane reviews are better than the normal systematic reviews published out there but within Cochrane reviews you do find quite poor quality Cochrane reviews’* (Participant 7)  *‘I mean I value them highly and just in general I would start with the assumption that it was good quality and obviously there’s variation in quality and I urge people not to assume that just because it’s a Cochrane review it’s good’* (Participant 6) |
| Perception that quality may be poorer in older reviews | *‘the newer one, is kind of better than the older Cochrane review’* (Participant 7) |
| **3. Culture and approaches** | |
| Cochrane & GD have similar attitudes towards evaluating, and appraising evidence | *‘I think their (Cochrane) processes are quite similar to the processes that we use, so if I know that there’s a Cochrane Review published I’ll definitely go to that as my first line’* (Participant 4) |
| Cochrane reviews routinely used to inform guideline development process | *‘Well for us in our guideline development Cochrane Reviews are key to all aspects of guideline development’* (Participant 2) |
| Some differences in methods (e.g. CR double data extraction but some GD not) | *‘I know, for Cochrane you will have at least two reviewers to go through everything together while obviously because of resource issue within (name of organisation) that isn’t 100%, so normally, we will have a second reviewer doing random cross-checks rather than everything’* (Participant 7) |
| Judgement part of guideline development process (but not part of CR process) | *‘When we’re developing a guideline, you know, we can’t just produce a guideline that says “There’s not enough evidence”, we have to be more pragmatic about it than that’.*  (Participant 8)  *‘In guideline development is what happens behind, really behind the scenes … all these beautiful and great, evidence profiles and .. tables, everybody carefully formulates them and they put them in annexes to guidelines but were they actually the basis on which the recommendations were crafted at the meetings is the catch and you know that’s hard to capture’* (Participant 6)  *‘when should* (name of organisation) *make expert opinion based recommendations because they need to do something and when should they say “Sorry, you know, just can’t say”* (Participant 6) |
| Cochrane and GDs may have different scopes/focus/drivers behind review questions | *‘There are people (referring to Cochrane reviewers) who will take a kind of broad view and put a lot of interventions together, and then do kind of analyses comparing among them. And that’s really the strategy that you need for a guideline. You’re interested in choosing among a set of treatment alternatives, knowing whether one treatment is better than nothing. And then there are a fair number of Cochrane reviews that look at one tiny little thing …well, it’s difficult for us to use that sort of thing in the guideline’* (Participant 3).  *‘There’s a historical problem there that the way Cochrane Collaboration started was driven by conditions, conditions with clinical questions in mind. And when you move onto guideline development at a national or international level obviously questions are slightly different’* (Participant 2) |
| Tensions between different perspectives and interests (e.g. academic/clinical/policy) | *‘They (Cochrane reviewers) perceive a particular question from more of an academic ground while within the guideline development group they tend to want to find answers that are more applicable on the ground, in clinical practice.’* (Participant 7) |
| Resources – different timeframes & sources of funding | *‘our main problem is that we want to get started and it takes so long to update or produce a new Cochrane review and by the time it’s done we’re past that stage anyway and we sort of do our own systematic review’* (Participant 8)  *‘we employ so many full time systematic reviewers churning out systematic reviews every six to eight weeks, so that doesn’t quite match up to, you know, the time frame of doing a Cochrane review’*  (Participant 7) |
| **4. Up-to-date evidence** | |
| CRs can be out of date | ‘*Is the right question there and in the library, and is it up-to-date?* (Participant 6).  ‘*the growth in this field is so rapid that, you know, if a Cochrane review is two years out of date, we’re going to end up redoing the searches and having to add studies to the analysis and things’* (Participant 3)  *‘Well see we would only search back a maximum of five years on a systematic review because after that we’re thinking it’s going to be out of date’*  (Participant 8) |
| Some confusion around dates of updates | *‘I think the one thing that we sometimes find is when Cochrane updates its reviews, it’s like which date do you use to put in your bibliography? Sometimes the, you know, it can say it’s from 2010 but you look at the actual body of the review and you think well actually the most substantive part is from 2007’* (Participant 1) |
| Delay in publications/ updates | *‘Well, it’s partly that things are unfunded but, you know, my experience of the last one that I submitted (name of group) was that it took seven months to get peer review on it, and ..then at that point, we’re asked to do the searches again because the searches…. So now we’ve got, you know, another four months delay or something while we update the searches and then we do all of the analysis. You know, it’s difficult to get a Cochrane review through in two years at this point’* (Participant 5 referring to delays in getting CRs published)  *‘One disadvantage is obviously it requires more time because it has to go through title registration, protocol review and final review, peer review stages. Because we are able to provide resources we can fast track it a little bit. But I mean that is a limitation, well that’s a limitation for other reviews as well if you were to start from scratch but obviously the Cochrane procedures can be more cumbersome’*  (Participant 2 referring to time it takes to get a Cochrane review completed) |
| **5. Methodological issues** | |
| Newer is better (newer CRs seen as methodologically better) | *‘the newer one, is kind of better than the older Cochrane review’* (Participant 7) |
| May be statistical issues (wrong data/statistical methods – barrier to use) | *‘sometimes I wasn’t clear about what correlations they used to adjust the standard deviations and I couldn’t find it inside the text’* (Participant 4) |
| Lack of clarity on which follow-up data used from papers | *‘I know they have a section on where they contacted the authors to get extra information, but sometimes it’s not clear how much extra information they did get’* (Participant 4) |
| Network meta-analysis, comparative analysis reviews | *‘I do think there’s need for more of these overviews of reviews and of reviews that, you know, incorporate a number of different interventions. I think that, you know, if you’re thinking about policy making, you’re not interested in one treatment’* (Participant 3) |
| GRADE (NICE have to use it Cochrane don’t) | *‘we are, we need to use grade as a quality appraisal’* (tool) (Participant 7) |
| Cochrane focus on RCTs – not always appropriate, particularly for public health | *‘obviously in public health the multi-component interventions, and population based interventions and exposures are far, far more complex than Cochrane’ … I mean, it’s* (referring to Cochrane) *working in that direction, certainly in the public health field’* (Participant 6)  *‘where Cochrane Collaboration is not currently addressing is to capture other types of research evidence, not necessarily related to interventions but for example about visibility, acceptability of the studies and so on which are becoming more and more important for guidelines’* (Participant 2) |
| GD want better facilities for sharing & reanalysing data from CRs | *‘I would like to see us get to a point where we’re sharing data. Where, you know, we’re entering information in a way that means when somebody has extracted fifty studies of drugs for schizophrenia, if we want to look at another question that isn’t answered in the way that they’ve analysed the data, we can go and just reanalyse the data’* (Participant 3)  *‘so if you’re combining scales and entering things using generic inverse scenarios, it’s then impossible to get back to the original data’* (Participant 3 referring to problems accessing data that has been entered into Cochrane review management software RevMan) |
| **6. Collaboration/communication** | |
| Good communication improves use of review | *‘I think it’s more about, you know, the communication and collaboration, so, you know, how can we share the information, I think that’s the problem at the moment, and it’s extremely difficult when you have so many different Cochrane groups and they all work differently, so, trying to establish that kind of relationship is quite difficult’* (Participant 7)  *‘If Cochrane database of systematic reviews was open access I think it would tremendously increase the impact’* (Participant 2) |
| Timing of communication important | *‘In fact they came up and talked to the group and gave them a presentation on what they’ve found and everything like that, they were very involved, it was very detailed and they were very generous and this is before it’s actually been published. It’s actually been really useful and worthwhile’* (Participant 1 referring to Cochrane reviewers)  *‘I tried to contact one of them, the actual contact reference that I, I didn’t manage to get in contact in time’* (Participant 4) |
| Dialogue/clear communication/negotiation important with appropriate persons | *‘we would send out information to the Cochrane review group that has done the reviews, our review questions and the reviews that we think would be appropriate to try and get some feedback about … whether any of those reviews are being updated or whether there’s any new reviews that we don’t know about in the pipeline.* (Participant 5) |
| Collaboration and positive engagement might help to speed things up | *‘We need to develop better working relationships with the groups that do them really and find a better way of communicating with them’*. (Participant 3 referring to communication with CRGs) |
| Close collaboration between WHO and certain Cochrane Groups (including funding for reviews/updates) | *And then the way we execute the guideline is we work very closely with the Cochrane (name of CRG)* (Participant 2)  *‘We provide the resources and they fast track or make sure that the Cochrane Reviews that will address our guidelines are conducted on a timely manner’* (Participant 2) |
| Formal links between CRG and guideline developers to promote use of CR | *‘One example where they have had a big impact is that we contact, we have contact with the Cochrane groups for each topic that we do and research them before we start the guidelines to see what they’ve got coming up and for them to look at our key questions to see if there’s anything that they can work on with that. With Cochrane, with chronic pain the Cochrane Group sort of flagged up that there were issues with the reporting methods in pain trials, computation methods with reporting. And so that had a big impact on how we appraised the evidence when we looked at what we were doing’.*  (Participant 8)  *‘we have occasionally been in contact with review groups in the past so that’s something that we’re just starting to do more comprehensively if you like’* (Participant 5 referring to efforts to set up links with CRGs) |
| GD experience problems communicating with CRGs | *‘We always try to but we don’t always get responses back from the authors’. (Participant 7 referring to communicating with review authors)*  *‘it’s extremely difficult when you have so many different Cochrane groups and they all work differently, so, trying to establish that kind of relationship is quite difficult’* (Participant 7) |
| Issues of ownership/authorship –recognition and reward | *‘I think there’s a concern, you know, on the Cochrane side, about how their work will be used and we need to find a way that, you know, Cochrane are confident that their work is being cited properly and represented accurately’* (Participant 3)  *‘So we’re currently now at the stage where we realised that a lot of existing reviews maybe nearly ten, exist and it would be inefficient to ignore the fact that they exist so we have been in contact with the Schizophrenia Cochrane Group and we’re sort of in the process of negotiating how we might use those existing reviews’* (Participant 5) |
